# Blood Levels of Different Trace Elements in the Endangered Iberian Lynx (Lynx pardinus) and Their Association with Endogenous and Exogenous Factors

**DOI:** 10.3390/ani16152442

**Published:** 2026-08-06

**Authors:** David Fernández-Casado, Elsa Rodríguez-Somoza, Ángel Portillo-Moreno, Alicia M. Carrillo-Heredero, Susana Sánchez-Cuerda, María Galán-Carrillo, Álvaro Guerrero, Salomé Martínez-Morcillo, María P. Míguez-Santiyán, Simone Bertini, Marcos Pérez-López, María J. Palacios, Francisco Soler-Rodríguez

**Affiliations:** 1 Toxicology Unit, Faculty of Veterinary Medicine, Av. de la Universidad s/n, 10003 Cáceres, Cáceres, Spain; davidfc@unex.es (D.F.-C.); elsars@unex.es (E.R.-S.); aportillom@unex.es (Á.P.-M.); martinezmorcillo@unex.es (S.M.-M.); mpmiguez@unex.es (M.P.M.-S.); solertox@unex.es (F.S.-R.); 2 Department of Veterinary Sciences, University of Parma, Strada del Taglio 10, 43126 Parma, Italy; aliciamaria.carrilloheredero@unipr.it (A.M.C.-H.); simone.bertini@unipr.it (S.B.); 3 Área del Medio Natural, Gestión Pública de Extremadura (GPEX), Junta de Extremadura, Carretera del Risco, s/n, 10181 Sierra de Fuentes, Cáceres, Spain; 4 AMUS (Acción por el Mundo Salvaje), Wildlife Rescue Center, Unnamed Rd, 06220 Villafranca de los Barros, Badajoz, Spain; 5 Dirección General del Medio Natural, Junta de Extremadura, Avenida Valhondo, s/n, Edificio Mérida III Milenio, 4ª Planta, 06800 Mérida, Badajoz, Spain

**Keywords:** metals, blood, Iberian lynx, exposure, biomonitoring

## Abstract

Trace elements are persistent environmental contaminants that can bioaccumulate and biomagnify through food webs, making wildlife valuable sentinels of ecosystem health. This study provides the first characterization of trace element concentrations in whole blood from free-ranging Iberian lynxes (*Lynx pardinus*) and evaluates the biological and environmental factors influencing their variability, including comparisons with captive individuals. A total of 229 blood samples collected in Extremadura (southwestern Spain) between 2018 and 2024 were analyzed for Cr, Mn, Cu, Zn, As, Se, Cd, Hg, Fe, and Pb. Overall, concentrations were within the ranges reported for other mammalian species, and no clinical evidence of adverse health effects was detected during routine veterinary examinations. Significant correlations were identified among several elements, particularly between Zn and Fe, with moderate associations between Mn and Zn and between Fe and Cu, suggesting common environmental sources and interconnected physiological regulation. Trace element concentrations were significantly influenced by age, sex, geographical area, and sampling period, especially for As and Se, reflecting differences in bioaccumulation, metabolism, and environmental exposure. Principal component analysis revealed a largely shared multielement profile, with no clear separation among populations or biological groups, indicating relatively homogeneous exposure despite moderate spatial and individual variability. These findings provide the first reference values for trace elements in Iberian lynx whole blood, establishing a baseline for biomonitoring, ecotoxicological assessment, and conservation of this threatened felid.

## Simple Summary

Trace element contamination may threaten wildlife health and ecosystem integrity. As an apex predator and one of the most threatened wild felids in Europe, the Iberian lynx is an important sentinel species for assessing environmental quality. This study presents the first evaluation of ten trace elements in blood samples collected from free-ranging and captive Iberian lynxes in southwestern Spain between 2018 and 2024. The objectives were to characterize current exposure levels and assess the influence of biological and environmental factors on trace element concentrations. Overall, concentrations were comparable to those reported in closely related felid species, and no clinical evidence of adverse health effects associated with trace element exposure was detected during veterinary examinations. Significant differences related to age, sex, geographical area, and sampling period were identified for several elements, indicating that both biological factors and environmental exposure contribute to the observed variability. Free-ranging lynxes generally exhibited higher concentrations of some trace elements than captive individuals, consistent with greater exposure to environmental sources. These findings provide the first reference values for trace elements in Iberian lynx blood and establish a baseline for future biomonitoring, environmental risk assessment, and conservation strategies supporting the long-term recovery of this emblematic species.

## 1. Introduction

Trace elements, including several metals and metalloids, are recognized as contaminants of increasing environmental concern because of their persistence and, for some elements, their potential for bioaccumulation and biomagnification, with consequent risks to wildlife and ecosystem health [1,2,3,4,5]. Essential trace elements, such as Cu, Fe, Mn, Se, and Zn, play fundamental roles in metabolic, enzymatic, and endocrine processes, whereas non-essential elements, including Cd, As, Hg, and Pb, have no known physiological function and may exert toxic effects even at low concentrations [6,7,8,9]. Wildlife may be exposed to trace elements through both natural geochemical processes [10] and anthropogenic activities, including mining, agriculture, and industrial emissions and discharges [11,12,13,14]. In addition, localized human activities such as hunting and fishing may further contribute to environmental contamination, particularly with Pb [15]. The toxicological effects of trace elements are element-specific and include hematological disorders, nephrotoxicity, neurotoxicity, genotoxicity, oxidative stress, and carcinogenesis [16,17,18].

Studies investigating trace element exposure in wildlife have used a wide range of biological matrices, including liver, kidney, brain, blood, hair, feathers, and bone, across diverse taxa ranging from aquatic organisms, such as fish, amphibians, and reptiles, to terrestrial vertebrates, including birds and mammals [19,20,21,22,23,24,25,26]. These investigations have also identified several species as valuable bioindicators of environmental contamination [27,28,29,30], facilitating the early detection of ecosystem disturbance and contributing to environmental monitoring programs [11].

The threatened Iberian lynx (*Lynx pardinus*), an apex predator endemic to the Iberian Peninsula, represents an especially suitable species for biomonitoring because of its high trophic position, marked dietary specialization based primarily on the European rabbit (*Oryctolagus cuniculus*), and strict habitat requirements [31,32]. Historical population declines were largely driven by disease outbreaks affecting its principal prey, together with other pressures, including habitat fragmentation and human activities [33,34,35]. Intensive conservation efforts implemented through four LIFE projects (Lince-Andalucía, Conservación y reintroducción del lince ibérico en Andalucía, Iberlince, and Lynxconnect, the latter currently ongoing) have substantially promoted population recovery, leading to the species being reclassified as “Vulnerable” in December 2024 [36,37,38].

From an ecotoxicological perspective, information on trace element exposure in the Iberian lynx remains limited. This represents an important knowledge gap, as apex predators are long-lived species occupying the highest trophic levels of food webs and are therefore particularly susceptible to the bioaccumulation and biomagnification of persistent environmental contaminants. Consequently, they are widely recognized as valuable sentinels of ecosystem health and environmental pollution. Assessing trace element exposure in apex predators can therefore contribute not only to improving species conservation and health management but also to advancing our understanding of contaminant dynamics and the potential risks to other species inhabiting the same ecosystems.

To date, studies on inorganic element contamination in the Iberian lynx have been restricted to destructive matrices, such as bone and liver [39], whereas non-destructive matrices, including whole blood, have not been investigated. A similar knowledge gap exists for other lynx species, including the Eurasian lynx (*Lynx lynx*) and the bobcat (*Lynx rufus*), for which recent ecotoxicological studies have likewise relied on liver samples [40,41,42]. Although destructive sampling provides valuable information on contaminant accumulation, it raises important ethical and ecological concerns, particularly for threatened species. In contrast, minimally invasive sampling offers an ethically sustainable approach for long-term biomonitoring, enabling repeated assessment of contaminant exposure while minimizing impacts on individuals and populations. Although blood may not always reflect contaminant burdens in target organs, its collection facilitates continuous health surveillance, temporal monitoring, and conservation-oriented research in free-ranging wildlife, especially elusive or protected species [43,44,45,46].

The present study provides the first assessment of metal and metalloid concentrations in whole blood from free-ranging Iberian lynxes. Concentrations of As, Cd, Cr, Cu, Fe, Hg, Mn, Pb, Se, and Zn were quantified, and the relationships among trace element concentrations, endogenous factors (age and sex), exogenous factors (geographical area), and temporal variation were investigated following approaches previously applied in wildlife ecotoxicology [47,48,49,50]. In addition, inorganic element concentrations were compared between free-ranging and captive individuals to assess differences associated with environmental exposure. The findings establish the first baseline reference values for trace elements in Iberian lynx whole blood and provide a valuable foundation for future biomonitoring, ecotoxicological risk assessment, and evidence-based conservation of this emblematic apex predator.

## 2. Materials and Methods

### 2.1. Study Area

Extremadura is located in the western Iberian Peninsula and covers an area of 41,634 km^2^. Despite its large territorial extent, the region has a population of 1,053,774 inhabitants, making it the least densely populated autonomous community in Spain. This low population density has favored the preservation of extensive natural areas with limited human disturbance, supporting a high diversity of plant and animal species. Consequently, Extremadura represents an important stronghold for several emblematic species, including the Iberian imperial eagle (*Aquila adalberti*), the threatened Iberian lynx, and the largest breeding populations of vultures in Spain.

**Figure 1:**
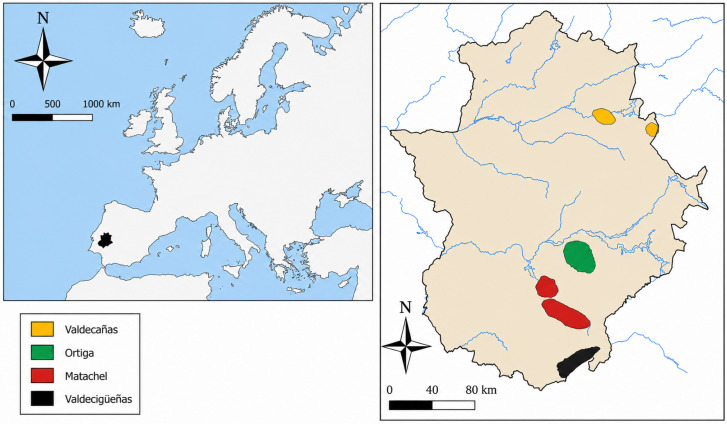
Distribution of the Iberian lynx in Extremadura.

The region encompasses several protected areas of high ecological value, including Monfragüe National Park, Tagus Internacional Natural Park, and La Siberia Biosphere Reserve. In addition, Extremadura contains 69 Special Protection Areas for Birds (SPAs; Zonas de Especial Protección para las Aves, ZEPA), which cover 26.15% of its territory, and is crossed by the Guadiana and Tagus river basins. These environmental characteristics provide suitable habitat for the Iberian lynx, whose current distribution in Extremadura is concentrated mainly in Matachel, Valdecigüeñas, the Ortiga River area, and the surroundings of the Valdecañas Reservoir within the Ibores region [36] (Figure 1).

- Matachel (38.4238, −5.7820) is located in southeastern Extremadura within a valley crossed by the Matachel River and bordered by the Sierra Grande de Hornachos. This area supports the largest Iberian lynx population in the region and is characterized by high biodiversity. The landscape consists of low-relief terrain with volcanic and clay-rich soils, predominantly used for cereal crops and vineyards. Anthropogenic pressures include three abandoned mines (Mina de Santa Marta, Zn–Pb; Mina Garandina, V; Mina Peñas Blancas, Cu–Ni). In addition, the A-66 motorway crosses the area and constitutes a known wildlife-vehicle collision hotspot, despite mitigation measures.
- Valdecigüeñas (38.0464, −5.9498) covers 37.34 km^2^ and is designated as a Site of Community Importance (SCI). It is bordered by the Pintado Reservoir and the Guadalcanal and Hinojales mountain ranges. The terrain is rugged, with quartzite, slate, and granite substrates. Historical mining activity includes Mina de la Jayona (Fe), currently designated as a Natural Monument. The area also hosts industrial infrastructure, including a galvanizing facility. This nucleus supports established Iberian lynx populations and a diverse assemblage of wildlife.
- Ortiga (38.9693, −5.9578) is located along the Ortiga River and its tributaries and forms part of the Natura 2000 network. The landscape is dominated by *dehesa* ecosystems (holm oak woodlands) and low quartzitic hills. Anthropogenic features include several abandoned mines (Mina San Nicolás, W; Mina Las Tejoneras, Cu; Mina El Pedregal, calcite) and an active granite quarry at Quintana de la Serena. The area is sparsely populated and is mainly used for agriculture, livestock production, and hunting activities.
- Valdecañas (39.7591, −5.6199) corresponds to the Valdecañas Reservoir, included within the Natura 2000 and Special Protection Area (SPA) networks and subject to wildlife management programs. It lies within the Tagus River basin and is characterized by sedimentary and granitic outcrops, with iron-rich formations currently under exploration for potential mining. The A-5 motorway crosses the area and represents a risk of wildlife–vehicle collisions affecting Iberian lynx.

### 2.2. Animals and Sample Collection

Between 2018 and 2024, a total of 172 Iberian lynx individuals were captured across the different study areas in Extremadura. A subset of individuals (n = 39) was recaptured in successive years, resulting in a total of 229 blood samples (n = 229). Captures were distributed among the previously described study areas and included individuals from different age classes.

Fieldwork was conducted within the framework of the LIFE Lynxconnect Project between October and January. The main objectives included health assessments (vital parameters, morphometrics, and body condition evaluation), GPS collar deployment for radio-tracking, and the collection of biological samples (blood, hair, and faeces) for toxicological, genetic, parasitological, and infectious disease analyses. Age determination of free-ranging individuals was based on long-term monitoring data generated within the project. All handling procedures were supervised by qualified veterinary staff, who were also responsible for the treatment of any injuries detected during capture events. All procedures were conducted in accordance with the approval granted by the Animal Ethics and Experimentation Committee of the University of Extremadura (Approval No. 50///2020; approved on 14 April 2020).

Standard live-capture methods using box traps were employed, followed by chemical immobilization using a combination of dexmedetomidine (0.015 mg/kg), midazolam (0.3 mg/kg), and ketamine (2.5 mg/kg). Approximately 4 mL of blood were collected from the jugular vein and transferred into BD Vacutainer^®^ (New York, NY, USA) lithium heparin tubes (4 mL) for toxicological analyses. Samples were transported under refrigerated conditions and stored at −80 °C within 2–4 days prior to laboratory processing.

A marked imbalance in sample distribution among study areas was observed, with Matachel accounting for the highest number of samples (n = 144), followed by Ortiga (n = 24), Valdecañas (n = 20), and Valdecigüeñas (n = 11).

In addition, 30 blood samples were obtained from captive individuals housed at the Iberian Lynx Breeding Centre as part of the ex situ conservation breeding program. Some of these individuals were subsequently released into the wild and continued to be monitored, allowing for additional sample collection during subsequent capture events.

### 2.3. Chemical Analyses

A total of ten metals and metalloids were analyzed: Cr, Mn, Cu, Zn, As, Se, Cd, Hg, Fe, and Pb. Whole-blood samples were processed at the Elemental and Molecular Analysis Laboratory of the University of Extremadura, accredited according to ISO 9001:2008 [51], following a previously validated in-house protocol. Approximately 400 µL of each sample were transferred into glass digestion tubes and subjected to acid digestion using 3 mL of a 3:1 mixture of 69% HNO_3_ and H_2_O_2_ in a Milestone Ultrawave microwave (Milestone Srl, Sorisole, Italy) digestion system. After digestion, samples were diluted to a final volume of 25 mL with ultrapure water.

Calibration curves were prepared using certified multielement standard solutions, and internal standards (Y and Rh, 400 µg/L each) were continuously introduced via a peristaltic pump. Elemental determinations were performed using an Agilent 7900 inductively coupled plasma mass spectrometer (ICP-MS; Agilent Technologies, Inc., Santa Clara, CA, USA) equipped with a fourth-generation octopole reaction system, operating in standard mode or in kinetic energy discrimination (KED) mode with He as collision gas.

Limits of quantification (LOQs) were 5 µg/L for Cr, Mn, Cu, As, Se, Cd, and Pb; 10 µg/L for Fe; 25 µg/L for Hg; and 50 µg/L for Zn. The limit of detection (LOD) and the LOQ were determined for each analytical batch by measuring elemental concentrations in six digestion blanks. The LOD was calculated as the mean blank concentration plus three times the corresponding standard deviation (LOD = mean blank concentration + 3 × SD), whereas the LOQ was defined as three times the LOD (LOQ = 3 × LOD). Instrumental performance was monitored daily (CeO/Ce < 2.5%, Ce^2+^/Ce < 3%, background < 1 cps). Method accuracy was assessed using the certified reference material Seronorm^®^ Trace Elements Whole Blood L-2 (SERO AS, Billingstad, Norway), yielding recoveries between 92% and 107% and coefficients of variation below 6.5% (Tables S1 and S2).

Fe was not analyzed during the 2020–2021 sampling period, whereas Cr was only included in the analytical protocol from that period onwards. Consequently, both elements were excluded from statistical comparisons among sampling periods.

### 2.4. Statistical Analyses

All statistical analyses were performed using GraphPad Prism 9.0.2 (GraphPad Software Inc., La Jolla, CA, USA). Descriptive statistics for trace element concentrations were expressed as mean ± standard deviation (SD), minimum (Min), 25th percentile (25th P), median, 75th percentile (75th P), maximum (Max), and range. Data normality was assessed using the Anderson–Darling, D’Agostino–Pearson, Shapiro–Wilk, and Kolmogorov–Smirnov tests.

Values below the LOQ were replaced with one-half of the limit of detection (1/2 LOD). Elements with a high proportion of censored data (Cr, Cd, and Hg; >60% below LOQ) were excluded from correlation analyses. In addition, Fe and Cr were excluded from comparisons among sampling periods due to incomplete datasets. The 2018/2019 sampling period was also excluded from temporal analyses because of the limited sample size (n = 4).

An alternative survival analysis was performed to evaluate the robustness of the 1/2 LOD substitution method, particularly for Pb. The results did not differ substantially from those of the original analysis and therefore did not justify changing the analytical approach for either Pb or the remaining trace elements.

Repeated samples from recaptured individuals were treated as independent observations in the descriptive analyses, as blood trace element concentrations are considered to primarily reflect recent exposure rather than long-term accumulation. However, the potential influence of individual identity was subsequently evaluated by including it as a random effect in the Generalized Linear Mixed Models (GLMMs) assessing the effects of endogenous and exogenous factors on blood trace element concentrations. Temporal dependence was also assessed to ensure that repeated sampling did not bias the results.

Given that data did not meet parametric assumptions, inferential analyses were conducted using non-parametric methods. Relationships between trace element concentrations were assessed using Spearman’s rank correlation coefficient. Correlation strength was interpreted according to Kalisińska et al. (2023) [52]. Statistical significance was set at *p* < 0.05. Differences among groups were evaluated using the Mann–Whitney U test (two groups) or the Kruskal–Wallis test (three or more groups).

The combined effects of sex, age, study area, and sampling period on trace element concentrations were evaluated using GLMM implemented in Jamovi 2.6 (GAMLj3 module). Trace element concentrations were treated as dependent variables, whereas sex (male/female), age class (kitten, juvenile, breeding adult), study area (Matachel, Ortiga, Valdecañas, Valdecigüeñas, and captivity), and sampling period (2018/2019–2023/2024) were included as fixed effects. Individual identity was included as a random effect (random intercept) to account for repeated measurements obtained from recaptured individuals across different sampling periods. Finally, principal component analysis (PCA) was performed using Jamovi 2.6 (jjPlots module) to explore multivariate patterns in trace element concentrations.

## 3. Results and Discussion

### 3.1. Metal and Metalloid Concentrations in Whole Blood

Descriptive statistics for trace element concentrations are presented in Table 1. All analyzed elements were detected in whole-blood samples, although Pb, Cr, Hg, and Cd were below the limit of quantification in 34.0%, 66.0%, 63.7%, and 69.9% of the samples, respectively. In contrast, the essential elements Zn, Cu, Fe, Mn, and Se, together with the metalloid As, were quantified in all samples. The ubiquitous detection of essential elements is consistent with their fundamental physiological roles and their interconnected functions in metabolic pathways [28].

To facilitate interspecific comparisons, mean trace element concentrations were compared with values previously reported for other mammalian species, including the caracal (*Caracal caracal*) [53], the Angora cat (*Felis catus*) [54], and the domestic dog (*Canis lupus familiaris*) [55] (Table 2).

Overall, mean concentrations followed the order Zn >> Cu = Fe > Se > As > Mn > Pb = Cr = Hg >> Cd. As expected, essential elements were present at the highest concentrations, whereas non-essential elements generally occurred at substantially lower concentrations, with the exception of As. The concentrations observed for each element and their toxicological significance are discussed below.

Although essential trace elements are indispensable for normal physiological function, excessive concentrations may adversely affect animal health. However, no reference intervals or toxicity thresholds have been established for trace elements in the blood of the Iberian lynx or other lynx species. Therefore, where appropriate, the present findings are discussed in relation to reference values reported for other mammalian taxa, acknowledging the inherent limitations associated with interspecific comparisons. These limitations underscore the need for further ecotoxicological studies in free-ranging carnivores to establish species-specific reference intervals and toxicity thresholds, thereby improving health assessment and conservation management of the Iberian lynx.

Zn was the most abundant element, with concentrations of 2729 ± 375.6 µg/L, remaining below the toxicity thresholds reported in horses (6000–15,000 µg/L), which have been associated with hemolytic anemia, renal failure, and gastrointestinal disorders [26,56]. The maximum recorded concentration (4962 µg/L) also remained below this toxicity range.

Mean Cu concentrations were 499.0 ± 109.0 µg/L, below the toxicity thresholds described in ruminants (1300 µg/L), in which chronic exposure may cause anemia, anorexia, and weakness, whereas acute exposure may induce diarrhea, dehydration, and hypovolemic shock [57,58]. A single juvenile male from Matachel sampled in 2019/20 reached a Cu concentration of 1684 µg/L without exhibiting clinical signs.

Fe concentrations were 459.4 ± 102.6 µg/L and did not exceed the toxicity thresholds (>3000 µg/L) established for mammals such as the mule deer (*Odocoileus hemionus*) and the black rhinoceros (*Diceros bicornis minor*) [59,60], above which clinical signs of intoxication such as lethargy, vomiting, and profuse diarrhea may occur [61]. The maximum concentration recorded (926.8 µg/L) likewise remained below this threshold.

Se concentrations averaged 354.7 ± 89.69 µg/L, remaining below toxicity thresholds reported for the mule deer (545 µg/L) and below concentrations associated with sublethal reproductive effects or overt toxicity in other taxa such as fish and birds [62,63,64,65,66]. A juvenile female from Matachel sampled in 2023/24 exhibited a Se concentration of 596.2 µg/L, slightly exceeding the proposed threshold, although no clinical signs of toxicity were observed.

Mean As concentrations were 146.3 ± 174.5 µg/L, exceeding the average values reported in healthy domestic cats (85 µg/L) [67]. The main symptoms associated with As intoxication include genetic alterations (DNA damage), behavioral changes, endocrine disruption, weakness, and diarrhea [67,68,69]. A juvenile female from Ortiga sampled in 2019/20 reached a maximum concentration of 1041 µg/L, markedly higher than reference values; nevertheless, the individual remained clinically healthy. The absence of species-specific toxicity thresholds for the Iberian lynx highlights the need for further investigation.

For Mn, the mean ± SD concentration was 27.82 ± 19.94 µg/L, considerably lower than the toxicity thresholds described in mammals such as the West Indian manatee (*Trichechus manatus*, 40,000–75,000 µg/L), in which anemia, serum biochemical alterations, renal insufficiency, and arrhythmias may occur above these levels [26,60]. The maximum concentration recorded was 295.6 µg/L.

For Pb, the mean ± SD concentration was 8.490 ± 14.86 µg/L, well below the toxicity thresholds established in mammals such as brown rat (*Rattus norvegicus*) and Japanese macaque (*Macaca fuscata*) (150–200 µg/L), above which hematological, neurological, and gastrointestinal effects may occur [6,70,71,72,73]. The maximum recorded value (169.4 µg/L) did not exceed this reference threshold, which was adopted in the absence of species-specific data. Under this criterion, several individuals fell within the proposed threshold range, although it is acknowledged that such thresholds are not universally applied in ecotoxicological studies. In approximately one-third of the samples, blood Pb concentrations could not be quantified.

Zn was the most abundant element, with a mean concentration of 2729 ± 375.6 µg/L. Both the mean and maximum concentration recorded (4962 µg/L) remained below the toxicity thresholds reported for horses (6000–15,000 µg/L), which have been associated with hemolytic anemia, renal failure, and gastrointestinal disorders [26,56].

Mean Cu concentration was 499.0 ± 109.0 µg/L, below the toxicity threshold reported for ruminants (1300 µg/L), where chronic exposure has been associated with anemia, anorexia, and weakness, and acute intoxication with diarrhea, dehydration, and hypovolemic shock [57,58]. However, one juvenile male from Matachel sampled during 2019/2020 exhibited a Cu concentration of 1684 µg/L without clinical evidence of toxicity.

The mean Fe concentration was 459.4 ± 102.6 µg/L, well below the toxicity threshold (>3000 µg/L) described for mammalian species such as the mule deer (*Odocoileus hemionus*) and the southern black rhinoceros (*Diceros bicornis minor*) [59,60]. Similarly, the highest concentration detected (926.8 µg/L) remained below this threshold, above which lethargy, vomiting, and severe diarrhea have been reported [61].

Mean Se concentration was 354.7 ± 89.69 µg/L, remaining below the toxicity threshold proposed for mule deer (545 µg/L) and below concentrations associated with sublethal reproductive impairment or overt toxicity in fish and birds [62,63,64,65,66]. Nevertheless, one juvenile female from Matachel sampled during 2023/2024 reached a concentration of 596.2 µg/L, slightly exceeding this reference value, although no clinical abnormalities were observed during veterinary examination.

Arsenic concentrations averaged 146.3 ± 174.5 µg/L, exceeding the mean value previously reported in clinically healthy domestic cats (85 µg/L) [67]. Exposure to As has been associated with DNA damage, behavioural alterations, endocrine disruption, weakness, and diarrhea [67,68,69]. The highest concentration (1041 µg/L) was detected in a juvenile female from Ortiga sampled during 2019/2020; despite this elevated value, the individual showed no clinical signs of intoxication. The absence of species-specific reference intervals or toxicity thresholds for the Iberian lynx precludes a definitive assessment of the toxicological significance of this finding.

The mean Mn concentration was 27.82 ± 19.94 µg/L, substantially lower than toxicity thresholds reported for the West Indian manatee (*Trichechus manatus*) (40,000–75,000 µg/L), above which anemia, alterations in serum biochemical parameters, renal dysfunction, and cardiac arrhythmias have been described [26,60]. The maximum concentration recorded was 295.6 µg/L.

Blood Pb concentrations averaged 8.490 ± 14.86 µg/L, remaining well below the toxicity thresholds reported for mammals such as the brown rat (*Rattus norvegicus*) and the Japanese macaque (*Macaca fuscata*) (150–200 µg/L), where hematological, neurological, and gastrointestinal effects have been documented [6,70,71,72,73]. The maximum concentration detected (169.4 µg/L) fell within this reference range, although these thresholds were adopted in the absence of species-specific data and are not consistently applied across ecotoxicological studies. Furthermore, Pb concentrations were below the limit of quantification in approximately one-third of the blood samples.

Mean Cr concentration was 6.075 ± 9.527 µg/L, remaining below the toxicity threshold (10 µg/L) reported for the small Indian mongoose (*Herpestes javanicus*) and the bonnet macaque (*Macaca radiata*), in which reproductive, endocrine, and other sublethal effects have been described [74,75,76]. Nevertheless, one breeding adult male from Matachel sampled during 2022/2023 exhibited a Cr concentration of 104.2 µg/L, greatly exceeding this reference value, although no clinical abnormalities were detected during veterinary examination.

Blood Hg concentrations averaged 5.795 ± 8.342 µg/L, substantially lower than the threshold reported for the Florida panther (*Puma concolor coryi*; formerly *Felis concolor coryi*) (500 µg/L), above which reproductive impairment has been documented [77,78]. Likewise, the maximum concentration detected (88.87 µg/L) remained well below this value.

Mean Cd concentration was 0.722 ± 0.326 µg/L, considerably lower than the toxicity threshold reported for mammals and birds such as the wood mouse (*Apodemus sylvaticus*) (26 µg/L), in which hematological alterations have been described [66,79]. The highest concentration recorded (1.560 µg/L) was also well below this threshold. Cadmium was the least frequently detected element, with quantifiable concentrations in only 30% of the samples.

The maximum values recorded for As, Hg, Pb, and Cr were substantially higher than the corresponding mean and median concentrations. Although these elevated values could be indicative of acute exposure to these trace elements, the good body condition and overall health status of the animals during the clinical examination, together with the absence of evident clinical signs, suggest that contamination of these samples cannot be ruled out.

It should be noted that the absence of clinical abnormalities during veterinary examinations does not exclude the possibility of chronic or sublethal effects associated with long-term exposure to, and accumulation of, trace elements. Such effects may remain undetectable during routine clinical assessments despite elevated blood concentrations.

Comparison with previously published data (Table 2) showed that Iberian lynxes (n = 229) generally exhibited lower trace element concentrations than caracals (*Caracal caracal*; n = 67) from South Africa, with the greatest differences observed for Mn, Zn, Cr, Hg, and Cd [53]. This pattern may reflect the greater environmental exposure of the South African population, which inhabits rapidly urbanizing coastal metropolitan areas influenced by both anthropogenic activities and marine-derived contaminant sources. In contrast, Angora cats (*Felis catus*; n = 30) from Türkiye showed lower Zn and Se concentrations than Iberian lynxes, whereas their reported Fe concentration (2440 ± 364.0 µg/L) greatly exceeded that observed in the present study (459.4 ± 102.6 µg/L) [54]. Similarly, domestic dogs (*Canis lupus familiaris*; n = 140) from Türkiye exhibited substantially lower As (21.02 ± 0.580 µg/L) and Hg (0.740 ± 0.220 µg/L) concentrations than Iberian lynxes (146.3 ± 174.5 µg/L and 5.795 ± 8.342 µg/L, respectively), while the remaining trace element concentrations were broadly comparable among species [55].

### 3.2. Correlation Between Metal and Metalloid Concentrations

**Figure 2:**
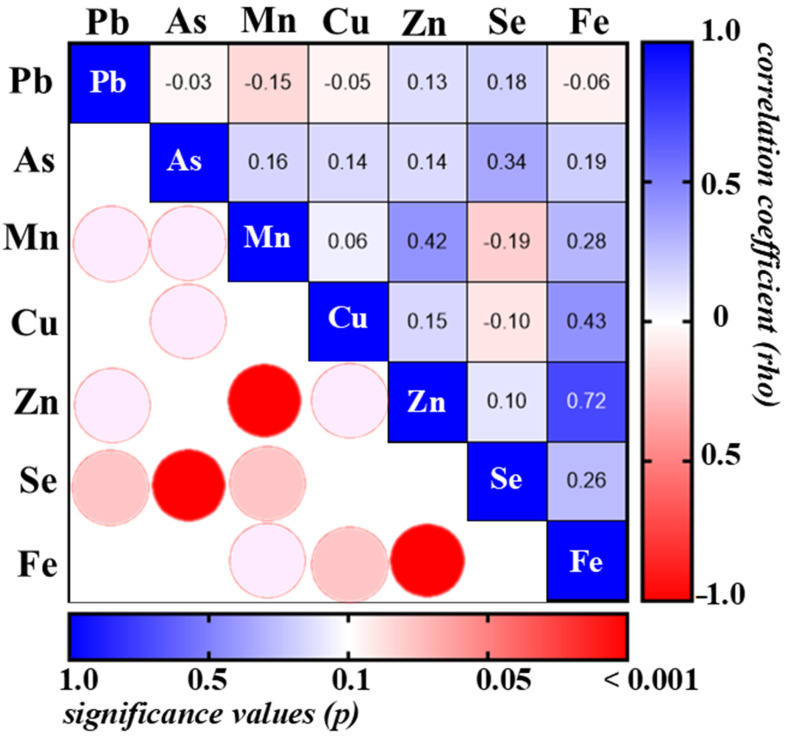
Spearman’s rank correlation matrix of metal concentrations in whole blood of the Iberian lynx. Correlation coefficients (rho) are shown in the upper triangle, while corresponding *p*-values are displayed in the lower triangle. Statistical significance was set at *p* < 0.05.

Relationships among trace element concentrations were investigated using Spearman’s rank correlation analysis (Figure 2).

Several statistically significant associations were identified. The strongest positive correlation was observed between Zn and Fe (*rho* = 0.72), whereas moderate positive correlations were detected between Mn and Zn (*rho* = 0.42) and between Fe and Cu (*rho* = 0.43). Weak positive correlations were also found between As and Se (*rho* = 0.34), Mn and Fe (*rho* = 0.28), and Se and Fe (*rho* = 0.26). All remaining pairwise associations were classified as very weak according to the criteria proposed by Kalisińska et al. (2023) [52].

To account for multiple comparisons, *p*-values were adjusted using the Benjamini–Hochberg false discovery rate (FDR) procedure, with statistical significance established at an adjusted *p*-value < 0.05. After FDR correction, the associations between Pb and Mn, As and Cu, and Mn and Fe were no longer statistically significant, suggesting that these correlations were likely attributable to Type I error resulting from multiple statistical testing.

Overall, the observed correlations indicate that several trace elements may exhibit common patterns of variation. These associations could reflect shared environmental exposure pathways, similar dietary sources, or physiological interactions affecting their intestinal absorption, transport, metabolism, and homeostatic regulation. Nevertheless, correlation analyses do not establish causal relationships, and the biological mechanisms underlying these associations require further investigation.

### 3.3. Influence of Endogenous (Age, Sex) and Exogenous (Sampling Period and Geographical Zone) Factors on Metal and Metalloid Concentrations

Among the endogenous variables evaluated, age and sex were included in the analyses, whereas body condition was excluded despite its recognized relevance in ecotoxicological studies. This decision was based on the limited variability observed within the study population, as 98% of the individuals exhibited a body condition score between 2.5 and 3.5, with 3/5 representing the optimal condition according to the scale used. In domestic cats, body condition is more commonly assessed using a nine-point scale, in which a score of 5/9 is considered ideal [80], corresponding approximately to a score of 3/5 in the present study. Body condition has previously been identified as an important covariate in studies evaluating trace element accumulation in tissues such as the liver [40]. However, those studies were based on post-mortem samples from animals suspected of having been diseased before death. Moreover, reduced body condition may not necessarily be a direct consequence of trace element toxicity but could instead promote contaminant accumulation [40].

The influence of endogenous and exogenous factors on blood trace element concentrations was evaluated using the Generalized Linear Mixed Model (GLMM), accounting for individual-level variability by including lynx identity as a random effect, thereby considering repeated measurements from recaptured individuals (Table 3). The mixed-effects models showed differences in explanatory power depending on the element analysed. The overall explanatory power of the mixed-effects models differed among trace elements, with the highest conditional *R*^2^ values obtained for Mn, Cu, As, and Se (conditional *R*^2^ = 0.738–0.892), whereas Pb and Zn showed lower overall model fit (conditional *R*^2^ = 0.341 and 0.547, respectively). Overall, the contribution of the fixed effects was lower than that of the random effects, highlighting substantial inter-individual variability in blood trace element concentrations.

Among the explanatory variables evaluated, study area was the only main effect that significantly influenced the concentration of a non-essential element, with significant differences detected for As (*p* = 0.015). In addition, sampling period and age significantly affected Se concentrations (*p* < 0.001 and *p* = 0.038, respectively). In contrast, no significant main effect of sex was detected for any of the analysed elements, although Zn showed a trend towards significance (*p* = 0.052).

Interactions among explanatory variables were particularly relevant for As and Se. For As, concentrations varied according to the combined effects of study area, sampling period, and age. Se, in turn, exhibited a pronounced interaction between study area and sampling period, indicating marked spatial and temporal heterogeneity. For Pb, only a single three-way interaction involving sex, sampling period, and age was detected, whereas no significant interactions were observed for Cu or Mn.

The proportion of variability attributable to individual identity differed markedly among trace elements. The highest intraclass correlation coefficients (ICCs) were obtained for Cu (0.720), As (0.625), and Se (0.312), indicating high individual consistency in the concentrations of these elements over time. In contrast, Pb showed relatively low repeatability (ICC = 0.171), whereas the random effect of individual identity was virtually negligible for Zn (Table S3).

Overall, the high individual variability observed for As and Cu, and to a lesser extent for Se, highlights the importance of including individual identity as a random effect in the models. A substantial proportion of the variation in blood trace element concentrations appears to be attributable to persistent individual differences, most likely associated with diet, space use, or intrinsic physiological characteristics of each animal.

In the case of Se, because whole blood primarily reflects recent exposure rather than long-term accumulation, the age-related differences observed are more likely to be associated with variation in diet, physiology, or trace element metabolism than with progressive bioaccumulation [39]. Potential mechanisms include developmental changes in intestinal absorption, homeostatic regulation of essential elements, and maturation of hepatic detoxification pathways [81,82]. The higher Se concentrations observed in adult individuals may reflect age-related differences in dietary exposure, trace element metabolism, or biotransformation capacity, together with developmental changes in the physiological regulation of this essential element [83].

Descriptive analyses by age class revealed a slight increase in mean Pb and Se concentrations with age (Table 4). Mean Pb concentrations increased from 6.502 ± 12.67 µg/L in kittens to 8.272 ± 12.49 µg/L in juveniles and 8.655 ± 16.51 µg/L in breeding adults. A similar pattern was observed for Se, with mean concentrations of 332.3 ± 82.26 µg/L, 356.7 ± 101.0 µg/L, and 361.0 ± 89.35 µg/L in kittens, juveniles, and breeding adults, respectively. In contrast, mean concentrations of the remaining trace elements showed little variation among age classes. The gradual increase in Pb concentrations may reflect cumulative differences in environmental exposure over time rather than bioaccumulation in blood itself. In the case of Se, the observed trend may additionally be associated with developmental changes in the homeostatic regulation of this essential element, as discussed above.

Descriptive analyses stratified by sex showed that mean Pb concentrations in males were almost twice those observed in females (8.500 ± 12.36 vs. 4.857 ± 4.560 µg/L, respectively) (Table 4). One possible explanation lies in the species’ spatial ecology. Male Iberian lynxes generally occupy larger home ranges and undertake longer movements associated with territorial behaviour and mate searching, potentially increasing their exposure to contaminated environments, including former mining areas and sites with intensive hunting activity. Their wider-ranging behaviour may also increase the likelihood of consuming prey originating from different areas, including game species or carrion containing Pb residues [84]. An alternative explanation is the transfer of Pb from females to their offspring during reproduction. Maternal transfer of Pb through lactation has been reported in other mammalian species [72,85], potentially reducing maternal Pb burdens. However, this mechanism is unlikely to have substantially influenced the present findings, as all blood samples were collected outside the breeding and lactation periods.

For As, sex-related differences in its metabolism, including the potential influence of estrogens on arsenic methylation, may partially explain the higher As concentrations observed in females [86]. However, given the limited information currently available on As toxicokinetics in wild mammals, additional studies are needed to clarify the mechanisms underlying these differences.

Mean concentrations of the remaining trace elements were broadly similar between sexes (Table 4), suggesting that sex-related physiological differences had only a limited influence on their circulating concentrations.

Temporal variation in trace element concentrations, the number of individuals sampled decreased from 45 in 2019/2020 to 31 in 2020/2021, before increasing progressively to 61 in 2023/2024 (Table 4). This trend is consistent with the continued recovery and expansion of the Iberian lynx population within the study area.

No significant temporal variation was detected for most trace elements. As was the only element showing an evident fluctuation in the descriptive analyses, with mean concentrations decreasing from 178.5 ± 232.2 µg/L in 2019/2020 to 88.67 ± 107.4 µg/L and 95.17 ± 159.7 µg/L in 2020/2021 and 2021/2022, respectively, before increasing again during the two most recent sampling periods. A similar, although less pronounced, pattern was observed for Pb, whose mean concentration declined from 16.45 ± 13.28 µg/L in 2019/2020 over the following years before stabilising at substantially lower values. These temporal fluctuations may reflect year-to-year variation in environmental exposure, prey availability, or movement patterns rather than sustained changes in contaminant accumulation.

**Figure 3:**
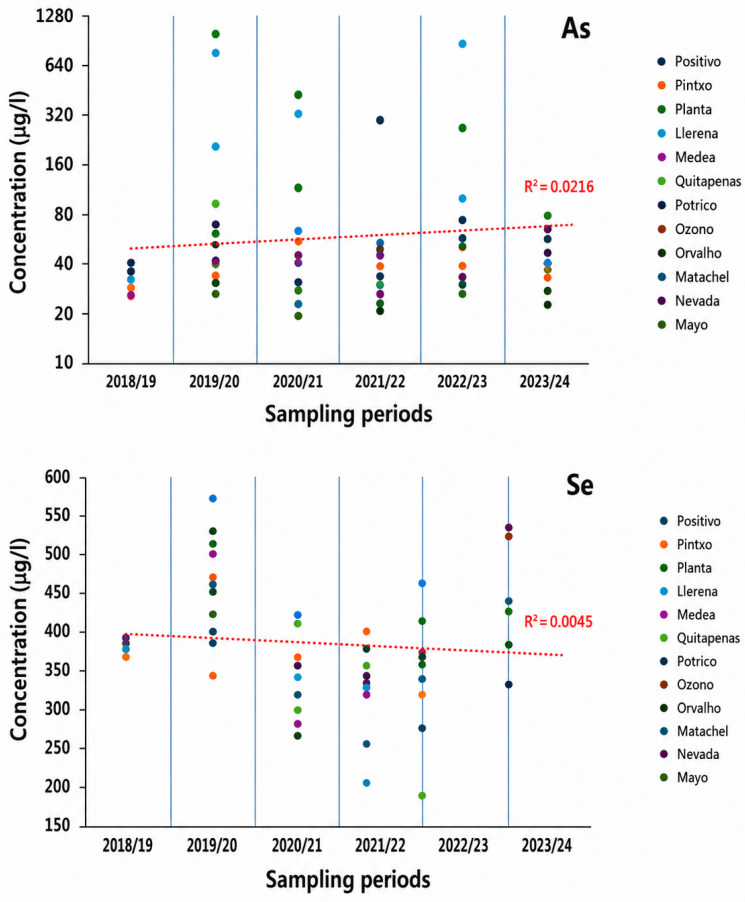
Temporal trends in whole blood As and Se concentrations of recaptured Iberian lynxes during successive sampling periods.

Among the analysed elements, only Se was significantly affected by sampling period (*p* < 0.001) (Table 3). Nevertheless, longitudinal analyses of individuals sampled during three or more consecutive monitoring periods did not reveal consistent increases in trace element concentrations over time (Figure 3). Although interannual variability was evident for both As and Se, the coefficients of determination were very low (*R*^2^ = 0.0045 and 0.0216, respectively), indicating that sampling year explained only a negligible proportion of the observed variability. These findings suggest that short-term fluctuations in environmental exposure, together with individual physiological and dietary differences, are more likely to account for the observed temporal patterns than progressive contaminant accumulation.

Spatial variation was also evaluated to assess differences in trace element concentrations among study areas. As concentrations were significantly higher in Matachel and Ortiga than in captive individuals. These differences most likely reflect variation in environmental exposure and diet and may indicate the presence of local exposure sources associated with the environmental characteristics of these areas. Unlike free-ranging lynxes, captive individuals receive standardised diets and are not exposed to the heterogeneous environmental sources of trace elements encountered in natural habitats [87]. Although no statistically significant spatial differences were detected for the remaining elements, free-ranging individuals generally exhibited slightly higher concentrations than captive animals, consistent with their broader environmental exposure. Pb represented the only exception to this pattern, with comparatively higher concentrations observed in individuals from Valdecigüeñas and in captive lynxes. This finding may be related to the origin of prey supplied in captivity, which frequently comes from hunting estates where game animals may contain Pb residues derived from lead-based ammunition. In contrast, the more diverse prey spectrum exploited by free-ranging Iberian lynxes across multiple territories may reduce sustained exposure to a single source of Pb contamination.

A study conducted by Thomason et al. [42] on hepatic trace element concentrations in bobcats (*Lynx rufus*) reported that geographic location exerted less influence on trace element burdens than dietary ecology and behavioural traits. In that study, the authors compared an apex predator with more diurnal and omnivorous mesocarnivores, highlighting the importance of ecological factors in determining contaminant exposure.

Mean concentrations of the remaining trace elements were broadly comparable among the different study areas.

### 3.4. Principal Component Analysis (PCA)

**Figure 4:**
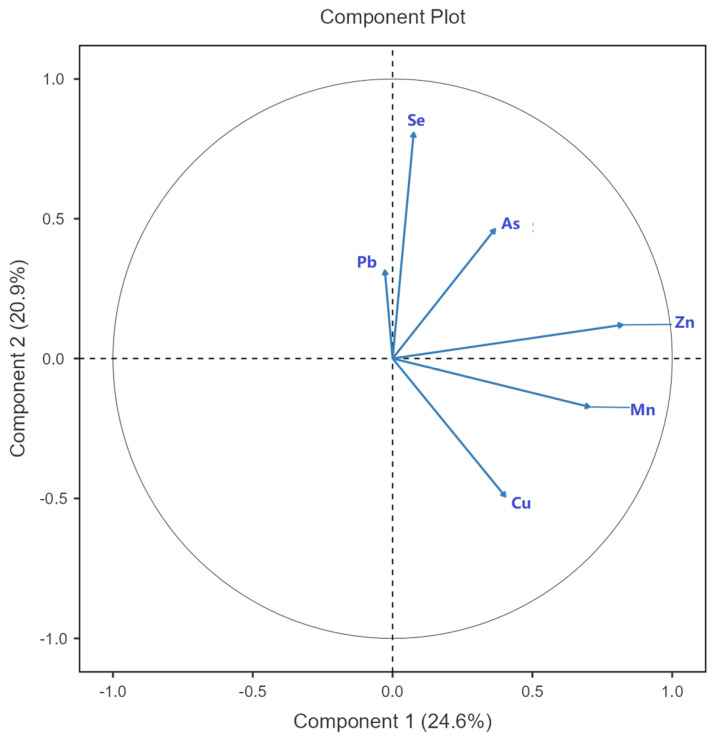
Distribution of trace elements in the different components generated by Principal Component Analysis (PCA).

Principal Component Analysis (PCA) showed that the first two principal components explained 45.5% of the total variance (Table S4). The first principal component (PC1) explained 24.6% of the variance and represented the main axis of variation in trace element concentrations, whereas the second principal component (PC2) explained 20.9% of the variance and primarily reflected differences in the relative contribution of essential and non-essential elements (Figure 4). Although this percentage is moderate, it is within the range commonly reported for ecological datasets characterized by high biological variability and therefore provides a useful summary of the multivariate structure.

**Figure 5:**
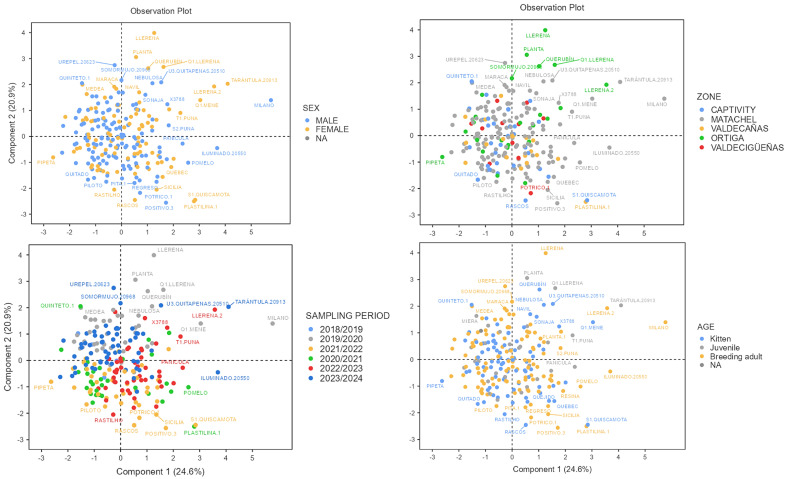
Distribution plots of samples according to sex, zone, sampling period and age based on the Principal Component Analysis (PCA).

Projection of individual Iberian lynxes onto the PC1–PC2 ordination space revealed no clear clustering according to study area (Figure 5), indicating that individuals from the different populations shared broadly similar multielement profiles. Nevertheless, slight spatial trends were apparent, possibly reflecting local variation in environmental conditions, dietary composition, or land use, including historical mining, agricultural, and livestock activities characteristic of the study areas.

Similarly, no distinct grouping was observed according to sex, sampling period, or age class (Figure 5), suggesting that these factors had only a limited influence on the overall multielement profile. However, kittens tended to occupy lower values along PC1 than breeding adults, indicating comparatively lower overall trace element burdens. Although whole blood mainly reflects recent exposure, this pattern may also be consistent with age-related differences in cumulative environmental exposure, diet, and physiological regulation, as discussed previously.

## 4. Conclusions

This study provides the first comprehensive characterization of ten trace elements in whole blood from free-ranging Iberian lynxes in Extremadura, based on a large sample set (n = 229). Blood concentrations of Pb, Mn, Cu, Zn, As, Se, Fe, Cr, Cd, and Hg were generally consistent with background levels reported for other mammals and closely related species, and no clinical evidence of trace element toxicity was detected during routine veterinary examinations. These findings suggest that Iberian lynxes inhabiting the study area are currently exposed to relatively low levels of these contaminants. Among the analyzed elements, As, and Se were significantly influenced by both endogenous and exogenous factors, including age, study area, and sampling period, highlighting the combined contribution of biological processes and environmental exposure to trace element variability. Free-ranging individuals generally exhibited higher As and Se concentrations than captive lynxes, consistent with greater environmental exposure under natural conditions. Although the concentrations detected do not currently indicate an immediate toxicological concern, continued biomonitoring is warranted, particularly in view of potential future changes in land use and industrial activity within the study region.

These findings establish the first reference values for trace elements in whole blood of the Iberian lynx and provide a valuable baseline for long-term biomonitoring, ecotoxicological assessment, and evidence-based conservation strategies. Continued research on apex predators such as the Iberian lynx will strengthen evidence-based conservation strategies for this threatened species while improving our understanding of contaminant dynamics and ecosystem health. As demonstrated in studies on other lynx species and apex predators, long-term biomonitoring of these sentinel species represents a valuable tool for assessing environmental quality and supporting biodiversity conservation [40,88,89].

## Figures and Tables

**Table 1:** Descriptive statistics of the overall results obtained from the analysis of metals and metalloids in whole blood of the Iberian lynx. Results are expressed in µg/L.

Metal and Metalloids	Mean ± SD	Min.	25% P.	Median	75% P.	Max.	Range	% <LOQ *	n
Pb	8.490 ± 14.86	0.800	1.000	6.200	9.355	169.4	168.6	34.0	229
As	146.3 ± 174.5	12.90	53.70	89.90	153.0	1041	1028	0	229
Mn	27.82 ± 19.94	11.30	20.45	25.80	31.70	295.6	284.3	0	229
Cu	499.0 ± 109.0	338.9	446.2	483.4	526.9	1684	1346	0	229
Zn	2729 ± 375.6	1813	2503	2679	2889	4962	3149	0	229
Se	354.7 ± 89.69	176.2	282.8	359.1	410.3	596.2	420.0	0	229
Cr	6.075 ± 9.527	0.000	1.000	3.900	8.100	104.2	104.2	66.0	179
Hg	5.795 ± 8.342	0.400	4.160	4.200	5.000	88.87	88.47	63.70	229
Fe	459.4 ± 102.6	331.4	408.8	437.4	476.2	926.8	595.4	0	50
Cd	0.722 ± 0.326	0.050	0.420	0.830	1.000	1.560	1.510	69.90	229

* Percentage of total samples with concentrations below the LOQ.

**Table 2:** Comparative summary of mean metals and metalloids concentrations (mean ± SD; µg/L) measured in the blood of three different species.

	Caracal (*Caracal caracal*)	Angora Domestic Cat (*Felis catus*)	Domestic Dog (*Canis lupus familiaris*)
Pb	3.000 ± 3.000	18.00 ± 1.700	12.37 ± 1.170
As	374.0 ± 838.0	-	21.02 ± 0.580
Mn	384.0 ± 179.0	34.00 ± 4.000	-
Cu	588.0 ± 566.0	593.0 ± 54.50	509.0 ± 6.730
Zn	5655 ± 5418	732.0 ± 71.50	3633 ± 74.45
Se	369.0 ± 155.0	270.0 ± 133.5	-
Cr	29.00 ± 76.00	27.00 ± 2.600	57.78 ± 1.110
Hg	45.00 ± 74.00	-	0.740 ± 0.220
Fe	-	2440 ± 364.0	763.7 ± 17.15
Cd	6.000 ± 14.00	-	0.190 ± 0.020
Sample size (n)	67	30	140
Location	South Africa	Turkey	Turkey
Reference	[53]	[54]	[55]

**Table 3:** Summary of the generalized linear mixed models (GLMMs) fitted for six trace elements. Each model included sex, age, study area, and sampling period as fixed effects, and individual identity as a random intercept to account for repeated measurements from recaptured individuals. Only factors with *p* < 0.05 were considered statistically significant.

Trace Elements	Type	R^2^	*p*-Value (Model)	n	Contributor Factors
Pb	Conditional	0.341	0.352	227	-
	Marginal	0.206	0.468		
As	Conditional	0.771	<0.001	227	Area
	Marginal	0.387	<0.001		
Mn	Conditional	0.892	0.004	227	-
	Marginal	0.185	0.157		
Cu	Conditional	0.781	0.049	227	-
	Marginal	0.218	0.049		
Zn	Conditional	0.547	<0.001	227	-
	Marginal	0.321	<0.001		
Se	Conditional	0.738	<0.001	227	Age, Sampling periods
	Marginal	0.619	<0.001		

**Table 4:** Descriptive analysis of the results obtained from the analysis of metals and metalloids in whole blood samples from Iberian lynx classified by sex, age, area and sampling periods. Results are expressed in µg/L.

		Mean ± SD					
		Pb	As	Mn	Cu	Zn	Se
Sex	Males	8.500 ± 12.36	121.6 ± 132.4	28.26 ± 25.26	502.0 ± 125.6	2692 ± 346.6	351.1 ± 82.28
	Females	4.857 ± 4.560	135.1 ± 154.4	27.39 ± 7.471	508.8 ± 83.30	2756 ± 415.2	338.8 ± 94.50
Age	Kittens	6.502 ± 12.67	137.7 ± 185.2	25.51 ± 7.336	500.2 ± 76.15	2704 ± 325.3	332.3 ± 82.26
	Juveniles	8.272 ± 12.49	198.8 ± 238.0	27.73 ± 11.70	469.2 ± 47.25	2783 ± 408.5	356.7 ± 101.0
	Adults	8.655 ± 16.51	138.2 ± 137.8	27.38 ± 9.506	499.3 ± 78.76	2716 ± 388.1	361.0 ± 89.35
Area	Matachel	8.211 ± 15.56	142.7 ± 157.2	29.10 ± 24.31	507.1 ± 122.5	2717 ± 404.6	360.9 ± 91.22
	Valdecañas	6.618 ± 7.985	115.8 ± 59.01	26.35 ± 8.002	508.9 ± 72.06	2763 ± 345.9	328.7 ± 78.69
	Ortiga	7.068 ± 6.530	334.1 ± 304.6	26.18 ± 9.822	465.0 ± 34.70	2686 ± 374.8	368.0 ± 89.41
	Valdecigüeñas	11.13 ± 18.31	73.67 ± 26.81	26.12 ± 6.225	482.7 ± 67.45	2768 ± 247.8	364.2 ± 89.53
	Captivity	11.25 ± 18.33	60.39 ± 43.98	24.62 ± 7.341	487.1 ± 102.1	2784 ± 290.8	328.5 ± 86.61
Sampling period	2019/20	16.45 ± 13.28	178.5 ± 232.2	28.68 ± 41.15	484.9 ± 191.6	2895 ± 480.2	426.6 ± 66.60
	2020/21	10.13 ± 15.87	88.67 ± 107.4	25.16 ± 8.590	494.6 ± 72.15	2703 ± 341.4	292.0 ± 66.66
	2021/22	5.720 ± 3.020	95.17 ± 159.7	26.58 ± 8.710	538.2 ± 107.2	2683 ± 315.0	282.7 ± 63.44
	2022/23	6.950 ± 25.20	175.7 ± 190.4	33.44 ± 8.750	488.7 ± 56.38	2813 ± 233.7	314.0 ± 66.52
	2023/24	4.530 ± 5.740	172.4 ± 140.2	25.26 ± 8.970	494.0 ± 50.36	2582 ± 369.2	415.2 ± 64.45

## Data Availability

Data is unavailable due to privacy or ethical restrictions.

## Supplementary Materials

The following supporting information can be downloaded at https://www.mdpi.com/article/10.3390/ani16152442/s1: Table S1: Summary of the quality control results obtained for Seronorm^®^ Trace Elements Whole Blood L-2 within the same analytical day (intra-day precision), together with a comparison with the manufacturer’s certified reference values. Table S2: Summary of the quality control results obtained for Seronorm^®^ Trace Elements Whole Blood L-2 (inter-day precision), together with a comparison with the manufacturer’s certified reference values. Table S3: Summary of the random-effects results from the generalized linear mixed models (GLMMs), including the variance components and intraclass correlation coefficients (ICCs) associated with individual identity as a random intercept accounting for repeated measurements from recaptured individuals. Table S4: Summary of the principal component analysis (PCA) evaluating the influence of endogenous and exogenous factors on blood trace element concentrations in the Iberian lynx.
