# Supplementary material for: Blood Levels of Different Trace Elements in the Endangered Iberian Lynx (Lynx pardinus) and Their Association with Endogenous and Exogenous Factors
Source: Animals (Basel). 2026 Aug 6;16(15):2442. doi: 10.3390/ani16152442 (PMC13464705; doi:10.3390/ani16152442)
Supplement: Supplementary file 1 [file animals-16-02442-s001.zip › animals-4411270-supplementary.pdf]

Supplementary Material

Table S1. Summary of the quality control results obtained for Seronorm® Trace Elements Whole Blood L-2 within the same analytical day (intra-day precision), together with a comparison with the manufacturer's certified reference values.

	**Seronorm® Trace Elements Whole Blood L-2**									
	**Quality control parameters**							**Intra-day precision**	**Manufacturer's**	
	**1**	**2**	**3**	**4**	**5**	**6**	**7**	**Mean±SD**	**Mean**	**Range**
**Mn (µg/l)**	28.91	30.37	27.53	27.91	26.99	27.66	26.94	28.04±1.219	29.9	17.9 - 41.9
**Cu (mg/l)**	1.23	1.28	1.30	1.34	1.37	1.34	1.30	1.309±0.046	1.3	0.79 - 1.87
**Zn (mg/l)**	5.82	6.07	6.02	6.10	6.14	5.96	6.03	6.020±0.105	6.5	5.8 - 7.2
**As (µg/l)**	13.15	13.30	14.09	14.28	14.42	13.94	13.93	13.87±0.478	14.3	8.5 - 20.1
**Se (µg/l)**	114.45	117.87	129.11	128.08	126.15	123.42	124.85	123.4±5.398	112.0	66 - 158
**Cd (µg/l)**	4.97	5.10	5.74	6.07	5.94	6.09	6.06	5.710±0.477	5.8	5.4 - 6.2
**Hg (µg/l)**	10.72	11.25	12.07	11.90	12.21	11.86	11.94	11.71±0.529	16.0	9.6 - 22.4
**Pb (µg/l)**	271.14	292.71	301.20	308.91	309.92	308.65	325.32	302.6±17.01	310.0	186 - 434

Table S2. Summary of the quality control results obtained for Seronorm® Trace Elements Whole Blood L-2 (inter-day precision), together with a comparison with the manufacturer's certified reference values.

	**Seronorm® Trace Elements Whole Blood L-2**							
	**Quality control parameters**					**Inter-day precision**	**Manufacturer's**	
	**Day 1**	**Day 2**	**Day 3**	**Day 4**	**Day 5**	**Mean±SD**	**Mean**	**Range**
**Cr (µg/l)**	-	8.6	14.7	6.39	7.84	9.383±3.662	10.2	9.23 – 15.2
**Mn (µg/l)**	28.04	31.4	40.3	24.02	27.83	30.32±6.161	29.9	17.9 - 41.9
**Cu (µg/l)**	1309	1285.6	1157.4	830.91	831.81	1083±236.8	1300	790 – 1870
**Zn (µg/l)**	6020	5837.0	5484.5	5126.0	5136.7	5521±404.3	6500	5800 - 7200
**As (µg/l)**	13.87	13.3	12.8	10.45	11.01	12.29±1.483	14.3	8.5 - 20.1
**Se (µg/l)**	123.4	103.0	77.3	72.33	82.73	91.75±21.19	112.0	66 - 158
**Cd (µg/l)**	5.710	6.3	6.0	4.80	1	4.762±2.177	5.8	5.4 - 6.2
**Hg (µg/l)**	11.71	20.1	4.2	14.86	5	11.17±6.714	16.0	9.6 - 22.4
**Pb (µg/l)**	302.6	300.0	276.7	301.55	325.86	301.3±17.41	310.0	186 - 434

Table S3. Summary of the random-effects results from the generalized linear mixed models (GLMMs), including the variance components and intraclass correlation coefficients (ICCs) associated with individual identity as a random intercept accounting for repeated measurements from recaptured individuals.

Trace elements	Groups*	Name	Variance	SD	ICC
Pb	Lynx (ID)	(Intercept)	40.30	6.350	0.171
	Residual		196.0	14.00	
As	Lynx (ID)	(Intercept)	14610	120.9	0.625
	Residual		8748	93.50	
Mn	Lynx (ID)	(Intercept)	411.4	20.28	0.867
	Residual		63.1	7.940	
Cu	Lynx (ID)	(Intercept)	9569	97.80	0.720
	Residual		3729	61.10	
Zn	Lynx (ID)	(Intercept)	41644	204.0	0.334
	Residual		83210	288.0	
Se	Lynx (ID)	(Intercept)	1103	33.20	0.312
	Residual		2435	49.30	

*Number of samples: 227; Number of groups: Lynx (ID) 187

Table S4. Summary of the principal component analysis (PCA) evaluating the influence of endogenous and exogenous factors on blood trace element concentrations in the Iberian lynx.

	Initial Eigenvalues			Extraction Sums of Squared Loadings		
**Component**	**Eigenvalue**	**% of Variance**	**Cumulative %**	**SS Loadings**	**% of Variance**	**Cumulative %**
**1**	1.477	24.6%	24.6%	1.477	24.6%	24.6%
**2**	1.254	20.9%	45.5%	1.254	20.9%	45.5%
**3**	1.070	17.8%	63.4%	-	-	-
**4**	0.921	15.3%	78.7%	-	-	-
**5**	0.811	13.5%	92.2%	-	-	-
**6**	0.467	7.8%	100.0%	-	-	-
